# CT Texture Analysis of Acute Ischemic Stroke: Prediction of Hemorrhagic Transformation After Thrombolysis

**DOI:** 10.3390/brainsci16060556

**Published:** 2026-05-23

**Authors:** Csaba Csutak, Diana Ursu, Andrei Lebovici, Manuela Lenghel, Andrei Roman, Calin Schiau, Adina Stan, Roxana Adelina Stefan, Mihnea-Ionut Nicoara, Paul-Andrei Stefan

**Affiliations:** 1 Department of Radiology and Medical Imaging, Faculty of Medicine, “Iuliu Hațieganu” University of Medicine and Pharmacy, 400012 Cluj-Napoca, Romania; csutakcsaba@yahoo.com (C.C.); andrei1079@yahoo.com (A.L.); lenghel.manuela@gmail.com (M.L.); andrei.roman678@gmail.com (A.R.); calin.schiau@yahoo.com (C.S.); 2 Department of Radiology, Emergency Clinical County Hospital Cluj-Napoca, 400006 Cluj-Napoca, Romania; mihnea.ionu.nicoara@elearn.umfcluj.ro (M.-I.N.); stefan_paul@ymail.com (P.-A.S.); 3 Department of Radiology, “Prof. Dr. Ion Chiricuță” Institute of Oncology, 400015 Cluj-Napoca, Romania; 4 Department of Neurosciences, Iuliu Hațieganu University of Medicine and Pharmacy, No. 8 Victor Babeș Street, 400012 Cluj-Napoca, Romania; dora.stan@umfcluj.ro; 5 Neurology Department, Emergency Clinical County Hospital, No. 43 Victor Babes Street, 400347 Cluj-Napoca, Romania; 6 Municipal Clinical Hospital of Cluj-Napoca, 3-5 Tabacarilor Street, 400139 Cluj-Napoca, Romania; roxanalupean92@gmail.com; 7 Department of Histology, “Iuliu Hațieganu” University of Medicine and Pharmacy, Victor Babes Street Number 8, 400347 Cluj-Napoca, Romania; 8 Faculty of Nursing and Health Sciences, “Iuliu Haţieganu” University of Medicine and Pharmacy, Victor Babes Street Number 8, 400347 Cluj-Napoca, Romania

**Keywords:** acute ischemic stroke, hemorrhagic transformation, non-contrast computed tomography, texture analysis, radiomics, thrombolysis, predictive modeling

## Abstract

**Highlights:**

**What are the main findings?**

- Baseline non-contrast CT texture features, specifically gray-level and run-length non-uniformity, showed exploratory discriminatory potential for characterizing tissue vulnerability to hemorrhagic transformation.

**What are the implications of the main findings?**

- Texture analysis may provide additional quantitative information that could support future bleeding risk stratification models.
- Further validation is required before CT radiomics can be integrated into acute stroke imaging workflows.

**Abstract:**

Background: Hemorrhagic transformation is a major complication after thrombolysis in acute ischemic stroke, and its risk assessment based on baseline non-contrast CT (NCCT) remains limited. This study aims to evaluate the role of CT-based texture analysis of the infarcted region on baseline NCCT in characterizing tissue vulnerability to hemorrhagic transformation. Methods: This retrospective single-center study included 50 patients with supratentorial acute ischemic stroke treated with thrombolysis. All patients underwent baseline NCCT and follow-up CT at 24 h and were divided into those with hemorrhagic transformation (*n* = 33) and those without (*n* = 17). Texture analysis was performed using MaZda software, and feature selection was conducted using the Fisher coefficient. Discriminatory ability was assessed using receiver operating characteristic analysis. Results: Gray-level and run-length non-uniformity features were significantly lower in patients with hemorrhagic transformation (*p* < 0.001). These parameters yielded AUCs of 0.94–0.97, highlighting their potential as exploratory imaging biomarkers. Conclusions: CT-based texture analysis of baseline NCCT may provide additional quantitative information associated with subsequent hemorrhagic transformation. However, given the small sample size, single-center design and absence of external validation, these findings should be interpreted as exploratory and hypothesis-generating.

## 1. Introduction

Hemorrhagic transformation (HT) remains one of the most feared complications following thrombolysis in patients with acute ischemic stroke (AIS), being associated with worse functional outcomes and increased mortality [1]. Despite extensive research, the ability to predict HT at the individual patient level remains limited, particularly in the early phase after symptom onset, when treatment decisions are made [2].

Non-contrast computed tomography (NCCT) represents the first-line imaging modality in AIS, primarily due to its widespread availability, rapid acquisition and established role in excluding intracranial hemorrhage and identifying early ischemic changes [3]. Conventional CT-based markers, including hypoattenuation, loss of gray–white matter differentiation and semi-quantitative scores such as the Alberta Stroke Program Early CT Score (ASPECTS), provide useful information regarding infarct extent but have limited predictive value for HT [4,5]. Similarly, combined clinical–imaging scores, such as the Hemorrhage After Thrombolysis (HAT) score and the SEDAN score (which incorporates blood sugar, early infarct signs, hyperdense artery sign, age, and National Institutes of Health Stroke Scale), improve risk stratification, but do not directly characterize the internal structural heterogeneity of the infarcted tissue [6,7].

More advanced CT-based approaches, including perfusion imaging and blood–brain barrier permeability assessment, have shown an improved ability to predict HT by probing the vascular substrate of reperfusion injury [8,9]. However, these techniques require contrast administration, dedicated acquisition protocols and specialized post-processing, which limit their routine clinical applicability in time-sensitive acute settings.

Radiomics and texture analysis have emerged as promising quantitative approaches that enable the extraction of imaging features reflecting spatial patterns of gray-level distribution and tissue heterogeneity beyond standard visual assessment [10,11]. Recent studies have suggested that radiomics features derived from baseline NCCT may provide predictive information regarding HT risk, indicating that even standard CT images may contain latent biomarkers of tissue vulnerability [12,13].

The aim of this study was to evaluate whether texture features extracted from the infarcted region on baseline NCCT are associated with subsequent HT in patients with AIS treated with thrombolysis and to assess their discriminatory ability.

## 2. Materials and Methods

### 2.1. Patients

This retrospective single-center observational study was conducted in accordance with the General Data Protection Regulation (GDPR) and approved by the institutional ethics committee of the “Iuliu Hațieganu” University of Medicine and Pharmacy Cluj-Napoca (registration number 50; date 11 March 2019). The requirement for informed consent was waived due to the retrospective nature of the study. Between January 2020 and December 2023, a total of 112 consecutive patients presenting to the emergency department with AIS and treated with intravenous thrombolysis were initially screened for eligibility.

Inclusion criteria were as follows:

- Supratentorial AIS confirmed on baseline NCCT;
- Involvement of the superficial cortical territory;
- Availability of baseline NCCT performed prior to thrombolysis;
- Follow-up CT performed at approximately 24 h after treatment, in accordance with institutional protocol;
- Visible infarct with a minimum diameter of 20 mm, allowing reliable placement of a region of interest for texture analysis.

Exclusion criteria consisted of:

- Infarcts located exclusively in deep structures (e.g., basal ganglia, thalamus);
- Severe motion or beam-hardening artifacts affecting image quality;
- Prior intracranial hemorrhage or pre-existing hemorrhagic lesions;
- Incomplete imaging data or missing follow-up examination.

**Figure 1:**
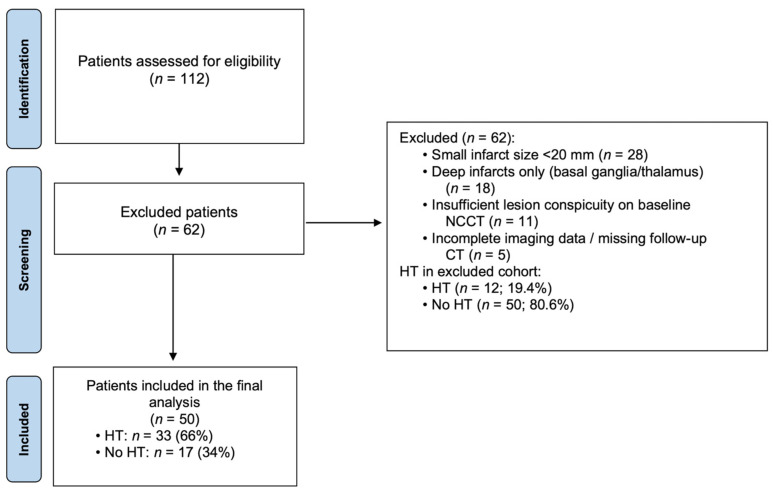
Flow diagram of patient selection illustrating inclusion criteria, reasons for exclusion, and distribution of hemorrhagic transformation (HT). HT—hemorrhagic transformation; NCCT—non-contrast computed tomography.

Of the 62 excluded patients, 28 were excluded because of small infarct size (<20 mm), 18 because of infarcts located exclusively in deep structures, 11 because of insufficient lesion conspicuity on baseline non-contrast CT for reliable ROI placement, and 5 because of incomplete imaging data or missing follow-up CT. Among the excluded cases, 12 (19.4%) developed hemorrhagic transformation and 50 (80.6%) did not. After applying these criteria, 50 patients were included in the final analysis. Patients were divided into two groups based on the presence or absence of HT on the 24 h follow-up CT. HT was defined according to the European Cooperative Acute Stroke Study (ECASS) classification, including both hemorrhagic infarction (HI1 and HI2) and parenchymal hematoma (PH1 and PH2) patterns. Of the included patients, 33 (66%) developed HT and 17 (34%) did not (Figure 1). The relatively higher proportion of hemorrhagic cases reflects the selection criteria requiring clearly identifiable infarct regions suitable for texture analysis, which favored larger infarcts with a higher baseline risk of transformation. This selection effect is further supported by the lower HT rate observed in the excluded cohort (19.4%) compared with the final analytical cohort (66%), indicating enrichment of the study population toward larger and more conspicuous infarcts.

The study cohort included 29 men and 21 women, with a mean age of 71 ± 9 years (range, 52–87 years). All patients received standard-dose intravenous thrombolysis (0.9 mg/kg recombinant tissue plasminogen activator), administered within the recommended therapeutic window according to current guidelines.

### 2.2. Texture Analysis Protocol

All CT examinations were performed using a multidetector CT scanner, according to institutional stroke imaging protocols (120 kVp, automatic tube current modulation, 5 mm slice thickness, soft tissue reconstruction kernel). The texture analysis workflow followed the conventional radiomics pipeline, including image segmentation, feature extraction, feature selection and statistical analysis, in line with previously described methodologies.

Image analysis was performed on a dedicated workstation (Advantage Workstation, version 4.7, General Electric Healthcare). All examinations were reviewed by two radiologists (with 6 and 10 years of experience in neuroradiology), both blinded to clinical outcomes. The two examiners established a consensus regarding the slice considered to be the most representative for the infarcted region on baseline imaging and, when applicable, for the hemorrhagic region on follow-up imaging. The resulting set of images was imported into the MaZda software (version 4.6), where regions of interest (ROIs) were defined for extraction of texture parameters.

**Figure 2:**
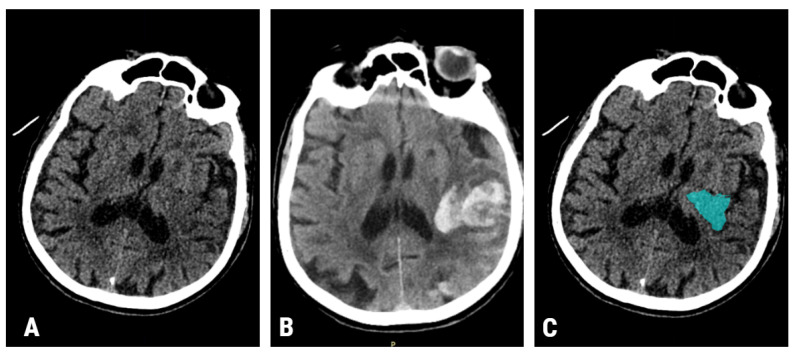
Representative case of a 72-year-old patient with acute left insular ischemic stroke and subsequent HT. (**A**) Baseline non-contrast CT demonstrating subtle hypoattenuation in the left insular region, consistent with early ischemic changes. (**B**) Follow-up CT at 24 h showing HT within the infarcted territory. (**C**) Region of interest (ROI) placed on the baseline CT, corresponding to the infarcted area that subsequently developed hemorrhage on follow-up imaging, used for texture analysis.

In patients who developed HT, the baseline and follow-up examinations were directly compared in order to identify the exact region of infarcted tissue that subsequently underwent HT. The region of interest was then placed on the baseline CT, specifically within the infarcted area corresponding to the location where hemorrhage was observed on the 24 h follow-up CT. This approach was used to ensure spatial correspondence between baseline and follow-up imaging; however, because the ROI location in HT cases was informed by the subsequent hemorrhagic area, we acknowledge it inherently introduces a retrospective localization bias into the sampling process (Figure 2).

In patients without HT, the ROI was manually placed on the infarcted area identified on the baseline CT, carefully encompassing the hypoattenuating region corresponding to ischemic tissue. All ROIs were two-dimensional and were visually verified to avoid inclusion of cerebrospinal fluid spaces, calcifications, or imaging artifacts.

All ROIs were placed on a single slice corresponding to the maximum lesion extent, ensuring consistency across the study cohort.

To reduce the impact of inter-scan variability, gray-level normalization was applied to each ROI using limitation of dynamics to μ ± 3σ, where μ represents the mean gray-level intensity and σ the standard deviation. This approach reduces the influence of extreme intensity values while preserving relative contrast within the region of interest.

To assess the reproducibility of the complete texture analysis workflow, interobserver and intraobserver agreement were evaluated in a randomly selected subset of 20 cases. For interobserver analysis, baseline NCCT images were independently re-segmented by a second radiologist (6 years of experience), blinded to the initial segmentation and clinical outcomes. For intraobserver analysis, the first radiologist repeated ROI placement after a 4-week interval, blinded to the initial contours. For each repeated segmentation, the full processing pipeline was reapplied, including ROI delineation, gray-level normalization using μ ± 3σ, feature extraction in MaZda, and calculation of the Fisher-selected texture parameters. Intraclass correlation coefficients (ICCs) were then calculated for the resulting feature values using a two-way random-effects model with absolute agreement. ICC values were interpreted according to established guidelines: poor < 0.5, moderate 0.5–0.75, good 0.75–0.9, and excellent > 0.9.

Texture features were extracted from multiple statistical domains, encompassing parameters derived from the gray-level histogram, absolute gradient, gray-level co-occurrence matrix (GLCM), run-length matrix (RLM), autoregressive model, and wavelet transform, reflecting different aspects of image intensity distribution and spatial heterogeneity. Gray-level discretization in MaZda is performed through image quantization, where the number of gray levels (Ng) is defined as Ng = 2^k^, with k representing the number of bits per pixel selected for each feature group. Different feature classes, such as GLCM- and RLM-derived parameters, may use distinct quantization levels depending on software settings. In the present study, texture features were extracted using the default quantization settings implemented in the software.

The features generated by the MaZda software include several hundred elements for each ROI. Given the high dimensionality of the extracted data, feature reduction techniques were applied in order to identify the most discriminative parameters and to enable subsequent statistical analysis. These techniques highlight subsets of features that maximize the separation between the analyzed groups while minimizing redundancy and classification error [14].

Feature selection was performed using the Fisher coefficient method. The Fisher coefficient (F) represents the ratio between interclass variance and intraclass variance, reflecting the ability of a parameter to discriminate between groups [15]. Higher Fisher values indicate greater discriminatory ability. Based on this approach, a subset of the most discriminative texture features was selected for analysis, consisting of the top-ranked parameters with the highest Fisher coefficients. This method allowed the identification of features with the strongest ability to differentiate between patients who developed HT and those who did not.

### 2.3. Statistical Analysis

Statistical analysis was performed using a commercially available dedicated software (MedCalc Software 23.5.0, Ostend, Belgium). The distribution of texture parameters was assessed using the Shapiro–Wilk test. As most parameters did not follow a normal distribution, comparisons between the two groups (patients with HT and those without) were performed using the Mann–Whitney U test. A *p*-value < 0.05 was considered statistically significant. To account for multiple comparisons in the univariate analysis, *p*-values were additionally adjusted using the Benjamini–Hochberg false discovery rate (FDR) procedure. Texture features that demonstrated statistically significant differences in the univariate analysis were evaluated using receiver operating characteristic (ROC) curve analysis. The area under the curve (AUC) was calculated for each parameter, along with 95% confidence intervals (CIs), to assess their discriminatory ability in predicting HT. Optimal cut-off values were determined using the Youden index, and corresponding sensitivity and specificity values were calculated.

## 3. Results

Fifty patients were included in the present study. Baseline clinical characteristics of the study population were analyzed descriptively. In the overall cohort of 112 patients, ASPECTS were 8–9 in 56 patients, 6–7 in 50 patients, 5 in 5 patients, and 4 in 1 patient. Most patients presented with mild-to-moderate stroke severity at baseline (*n* = 100), while 12 patients had moderate-to-severe NIHSS scores. Within the final analytical cohort, hemorrhagic transformation was more frequently observed in patients with lower ASPECTS. Specifically, HT occurred in 22 of 37 patients with ASPECTS 8–9, 10 of 12 patients with ASPECTS 6–7, and in the single patient with ASPECTS 5. Baseline demographic and clinical characteristics were not systematically stratified between HT and non-HT groups due to the retrospective design and incomplete availability of all variables.

Following texture feature extraction, the MaZda software automatically selected the most discriminative parameters using the Fisher coefficient method. The resulting features and their corresponding Fisher coefficients are presented in Table 1. Interobserver agreement for the selected texture features was good to excellent, with ICC values ranging from 0.79 to 0.92, with most parameters demonstrating values above 0.85. Intraobserver reproducibility was consistently high, with ICC values ranging from 0.86 to 0.96, indicating excellent repeatability of ROI placement and feature extraction. Because repeated ROI delineation and full feature extraction were performed for each observer, these ICC values reflect reproducibility of the complete segmentation and texture extraction workflow rather than feature recalculation from identical ROIs.

The selected parameters were predominantly derived from the run-length matrix (RLNonUni) and gray-level non-uniformity (GLevNonU) features, computed across multiple spatial directions (horizontal, vertical, 45°, and 135°). In contrast, parameters derived from the co-occurrence matrix demonstrated substantially lower Fisher coefficients.

After correction for multiple comparisons using the Benjamini–Hochberg false discovery rate procedure, all selected texture features remained statistically significant (adjusted *p* < 0.05). The comparison of texture parameters between patients with and without HT is presented in Table 2.

All features selected using the Fisher coefficient method showed statistically significant differences between the two groups. Specifically, parameters derived from the run-length matrix (RLNonUni) and gray-level non-uniformity (GLevNonU) were consistently lower in patients who developed HT across all evaluated directions (all *p* < 0.001). Co-occurrence matrix-derived features (S(4, 0)Correlat and S(3, 0)Correlat) also differed significantly between groups, (*p* = 0.02 and *p* = 0.04, respectively). However, their overall discriminatory ability remained limited.

The discriminatory performance of the selected texture parameters was evaluated using receiver operating characteristic (ROC) curve analysis, as summarized in Table 3.

**Figure 3:**
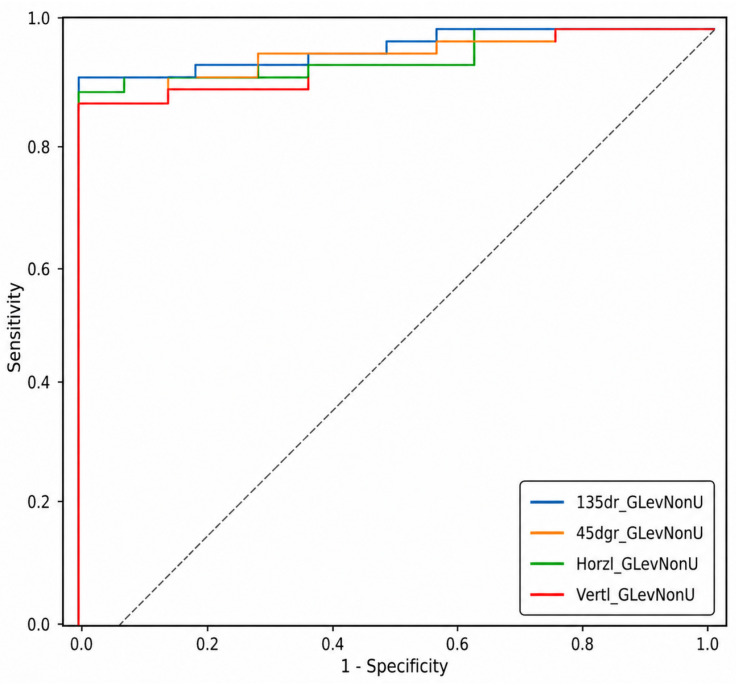
Receiver operating characteristic curves of the best-performing individual texture features for predicting hemorrhagic transformation.

Texture features derived from run-length matrix and gray-level non-uniformity showed high discriminatory performance in this exploratory cohort, with area under the curve (AUC) values ranging from 0.94 to 0.97. Among these, 135dr_RLNonUni and 45dgr_RLNonUni achieved the highest values, exceeding 0.96, followed closely by the remaining RLNonUni and GLevNonU features across all evaluated directions. These parameters also showed high sensitivity and specificity values at the optimal cut-off points determined using the Youden index. In contrast, co-occurrence matrix-derived features (S(4, 0)Correlat and S(3, 0)Correlat) yielded AUC values below 0.70, reflecting a lower discriminatory ability compared to the other texture parameters (Figure 3).

These findings indicate substantial redundancy between the top-ranked features, suggesting that several parameters capture overlapping aspects of lesion heterogeneity and gray-level distribution. Accordingly, multivariate regression modeling was not considered informative, and the study focused exclusively on the univariate discriminatory ability of individual texture parameters.

## 4. Discussion

The present study evaluated the potential role of CT-based texture analysis in exploring tissue vulnerability to HT in patients with AIS treated with thrombolysis. The results suggest that texture parameters extracted from the infarcted region on baseline NCCT differed significantly between patients who subsequently develop HT and those who do not. Features derived from gray-level non-uniformity and run-length matrix analysis showed high discriminatory performance, with AUC values exceeding 0.94. These findings suggest that baseline CT may contain quantifiable imaging patterns associated with HT risk that are not accessible through conventional visual assessment, a limitation consistently reported in previous studies investigating imaging biomarkers of HT after thrombolysis [2,4].

The most discriminative texture features in our study were run-length non-uniformity and gray-level non-uniformity, both of which showed lower values in patients who subsequently developed HT. According to the Image Biomarker Standardization Initiative, run-length non-uniformity decreases when run lengths are more evenly distributed, whereas gray-level non-uniformity decreases when gray-level intensities are more uniformly distributed within the region of interest [11,16]. In the present cohort, these findings suggest that infarcted regions prone to HT exhibited a more uniform distribution of attenuation values on baseline CT, rather than being dominated by a limited range of gray levels or extended homogeneous structures. From a pathophysiological perspective, such a pattern may reflect a spatially intermixed tissue composition, consistent with the complex microenvironment associated with ischemic injury and subsequent HT, including blood–brain barrier disruption and reperfusion-related changes [1]. The potential relevance of these features as imaging biomarkers is corroborated by recent literature demonstrating that both run-length non-uniformity and gray-level non-uniformity serve as informative features in independent radiomics evaluations for HT [12,17].

In contrast to run-length and gray-level non-uniformity features, parameters derived from the gray-level co-occurrence matrix, namely correlation features, demonstrated limited discriminatory performance in our study. Although these parameters reached statistical significance in univariate analysis, their AUC values remained below 0.70, indicating poor discriminatory ability in differentiating between patients with and without HT. Correlation features quantify the linear dependency between gray-level values of neighboring pixels, reflecting local spatial relationships within the image. The relatively weak performance of these parameters suggests that such pairwise relationships may not adequately capture the complex spatial heterogeneity associated with HT. This observation is consistent with previous texture analysis studies, which have shown that higher-order statistical features, such as those derived from run-length and gray-level non-uniformity matrices, may provide superior discriminatory performance compared to co-occurrence-based parameters [18]. Furthermore, because the top-ranked texture parameters demonstrated substantial mathematical and biological overlap, any multivariable modeling in this limited exploratory cohort primarily reflected feature redundancy rather than independent predictive contributions. Given the extreme multicollinearity inherent to these overlapping radiomic features, we intentionally restricted our final analysis to univariate feature exploration to avoid presenting an unstable or artificially inflated prediction model.

Several CT-based approaches have previously been explored for estimating the risk of HT after reperfusion therapy in AIS. While conventional NCCT markers and established clinical-imaging scores, such as the HAT and SEDAN scores, provide valuable bedside risk assessment, they do not directly characterize the internal structural heterogeneity of the infarcted tissue [2,4,6,7]. Furthermore, standard clinical and imaging features, including baseline stroke severity and infarct extent, are known to strongly influence tissue outcomes. Within our final cohort, the observed trend of a higher rate of hemorrhagic transformation among patients with lower baseline ASPECTS aligns with the known association between a larger infarct burden and increased risk of hemorrhagic transformation.

However, the present study was not designed to integrate clinical variables for multivariable risk stratification, and therefore a direct comparison with established clinical scores such as HAT and SEDAN was not feasible. Consequently, the results should be interpreted as reflecting the isolated, exploratory contribution of imaging-based texture features. Future studies should evaluate combined clinical-radiomics approaches to determine the incremental value of texture analysis beyond established clinical parameters.

These observations underscore that texture analysis captures a discriminatory signal primarily related to the internal distribution of attenuation values, reflecting latent microstructural organization rather than global lesion extent. Consequently, radiomics may be viewed as a complementary tool in acute stroke imaging. Rather than replacing established risk stratification tools, this quantitative assessment of tissue heterogeneity could eventually augment them by providing objective insights into tissue vulnerability.

When compared to previous NCCT-based radiomics literature, the present analysis utilized a more focused feature selection strategy in a smaller cohort. Ren et al. included 517 patients from seven hospitals, extracted 1037 radiomics features, retained 778 reproducible features after ICC filtering, and finally selected 12 radiomics features using recursive feature elimination; their radiomics model achieved AUCs of 0.922 in the internal validation cohort and 0.883 in the external validation cohort, while the combined clinical-radiomics model reached AUCs of 0.950 and 0.942, respectively [12]. Zhang et al. included 180 patients from two centers, extracted 851 radiomics features, retained 706 after ICC screening, and selected six features using LASSO and 5-fold cross-validation; their radiomics model achieved AUCs of 0.813 in the training cohort and 0.898 in the validation cohort, whereas the clinical-radiomics model reached 0.876 and 0.957, respectively [13]. In contrast, the present study included a smaller single-center cohort and focused on a limited set of Fisher-selected texture descriptors, mainly gray-level and run-length non-uniformity features, without constructing a complex machine-learning signature. While the high AUC values observed for our individual parameters are comparable to the apparent discriminatory ability reported in those larger studies, they must be interpreted more cautiously because of the smaller sample size, single-center design and absence of external validation.

Several limitations of the present study should be acknowledged. First, this was a single-center retrospective study with a relatively limited sample size, particularly within the non-HT group, which affects the generalizability of the results and increases the risk of overfitting. This is compounded by a significant selection bias: our strict inclusion criteria required visually conspicuous infarcts of at least 20 mm to allow reliable ROI placement. Consequently, the final analytical cohort was enriched with larger, more severe infarcts. Because excluded patients had a substantially lower HT rate, our cohort does not represent the broader, standard post-thrombolysis population. This spectrum enrichment artificially inflated the HT rate to 66% and may have increased the separability between groups, limiting generalizability, especially for patients with smaller or subtler infarcts. As a result, the observed discriminatory ability may be optimistic and should be interpreted with caution, underscoring the absolute need for larger, multicenter validation studies. In addition, the absence of an external validation cohort limits the robustness of the exploratory findings and raises the possibility of overfitting, a well-recognized issue in radiomics studies [10,11]. Second, texture analysis was performed using two-dimensional regions of interest placed on a single representative slice, rather than full volumetric segmentation. Although this approach improves reproducibility and reflects a more practical clinical workflow, it may not fully capture the complete spatial heterogeneity of the infarcted tissue. Third, in patients with HT, the region of interest on baseline CT was retrospectively localized using the site of subsequent hemorrhage on follow-up CT. This represents a major source of potential bias because the analysis sampled tissue already known to undergo hemorrhagic transformation. Therefore, the observed discriminatory ability may overestimate performance in a true prospective setting, where the future hemorrhagic region is unknown at baseline. Furthermore, the selection of the representative slice and ROI was based on visual assessment by experienced radiologists, which may introduce a degree of operator dependency and potential selection bias. Although a consensus approach was used, this process remains partly subjective and may influence the extracted texture features. At the same time, in acute ischemic stroke on baseline NCCT, infarct delineation is often challenging due to low lesion conspicuity, particularly in early stages. Under these conditions, manual selection remains a pragmatic approach for initial exploratory analyses. Automated or semi-automated methods may help reduce operator dependency and improve reproducibility in future studies. Another limitation of the present study is the absence of a detailed comparison of baseline demographic and clinical characteristics between the HT and non-HT groups. As a result, potential confounding effects of age, sex, comorbidities, and infarct size could not be formally assessed.

Finally, the biological interpretation of texture features remains indirect. Although parameters such as gray-level and run-length non-uniformity are thought to reflect tissue heterogeneity, their exact correspondence with specific histopathological processes cannot be established in the absence of direct tissue correlation, which represents a general limitation of radiomics-based analyses [10,11]. Regarding the single-slice 2D segmentation strategy noted above, previous studies using the filtration-histogram technique have directly compared single-slice and multislice/volumetric approaches on computed tomography in primary colorectal cancer for prognostic assessment [19], as well as on MRI in gliomas for differentiation between IDH-mutant and IDH-wildtype tumors [20]. Notably, those studies showed that single-slice analysis remained informative for outcomes in colorectal cancer [20] and for molecular stratification in gliomas [19], with performance comparable to multislice or volumetric methods. Therefore, the incremental benefit of full volumetric analysis remains uncertain, particularly given the additional time required for segmentation in time-sensitive stroke scenarios and the potentially greater operator-dependent variability associated with multislice or three-dimensional contouring. An additional key limitation relates to the reproducibility of texture feature extraction across software platforms. Although the analyzed parameters (run-length non-uniformity and gray-level non-uniformity) correspond to feature families described by the Image Biomarker Standardization Initiative (IBSI) [11], feature extraction was performed using MaZda software (version 4.6), which predates full IBSI standardization. As a result, the absolute values of the extracted features may be software-dependent, potentially limiting direct comparability with studies using different radiomics pipelines.

In the present study, the endpoint included all radiographic hemorrhagic transformation subtypes according to the ECASS classification, encompassing both hemorrhagic infarction (HI) and parenchymal hemorrhage (PH). This choice was intentional, as early radiographic HT—irrespective of subtype—may reflect underlying microvascular injury and blood–brain barrier disruption, processes that precede clinically significant hemorrhagic complications. From this perspective, the inclusion of all HT subtypes allows for the exploration of early tissue vulnerability rather than only advanced hemorrhagic events. However, we acknowledge that not all HT subtypes carry the same clinical significance, with PH and symptomatic intracranial hemorrhage being more strongly associated with adverse outcomes. Due to the limited sample size, subgroup analysis was not feasible. Future studies with larger cohorts should specifically assess the capacity of individual texture features to differentiate between clinically relevant hemorrhagic subtypes.

Future studies should focus on the standardization of image acquisition, preprocessing and feature extraction pipelines, in order to improve reproducibility and facilitate the potential clinical translation of CT-based radiomics approaches [21,22]. Another important direction is the development of integrated models combining radiomics with established clinical and imaging parameters, since hybrid approaches may provide more comprehensive risk stratification than imaging features alone [12,23]. In addition, prospective studies are needed to determine whether baseline NCCT radiomics can provide incremental value over conventional scores and whether these parameters remain robust across different scanners, reconstruction settings and patient populations. Ultimately, the clinical utility of such approaches will depend not only on their discriminatory ability, but also on methodological transparency, external validation and seamless integration into routine acute stroke workflows [21,22,23]. If confirmed by future studies, the primary clinical value of these texture biomarkers may lie in guiding post-thrombolysis management rather than altering the initial decision to treat. Identifying patients at a potential higher risk for bleeding on baseline imaging could justify closer neurological observation and an earlier threshold for follow-up scans. Consequently, NCCT radiomics could eventually serve as a practical tool for optimizing early patient care in the stroke unit.

## 5. Conclusions

CT-based texture analysis of baseline non-contrast CT imaging may provide additional quantitative information for characterizing tissue vulnerability to hemorrhagic transformation prior to thrombolysis. In particular, gray-level and run-length non-uniformity features demonstrated high discriminatory ability, suggesting that subtle spatial patterns within the infarcted tissue may reflect underlying microstructural heterogeneity not appreciable on conventional visual assessment. However, given the single-center design, limited sample size and absence of external validation, these findings should be interpreted with caution and considered hypothesis-generating. Further large-scale, multicenter studies are required to confirm the reproducibility and potential clinical applicability of these imaging biomarkers.

## Figures and Tables

**Table 1:** Texture features selected using the Fisher coefficient method.

Feature	F Value
135dr_RLNonUni	6.23
45dgr_RLNonUni	6.14
Vertl_GLevNonU	6.01
Horzl_RLNonUni	5.92
Horzl_GLevNonU	5.72
45dgr_GLevNonU	5.64
135dr_GLevNonU	5.62
Vertl_RLNonUni	5.50
S(4, 0)Correlat	0.59
S(3, 0)Correlat	0.58

F, Fisher coefficient; RLNonUni, run-length non-uniformity; GLevNonU, gray-level non-uniformity; S(4, 0)Correlat, gray-level co-occurrence matrix correlation (offset 4, 0); S(3, 0)Correlat, gray-level co-occurrence matrix correlation (offset 3, 0); Horzl, horizontal direction; Vertl, vertical direction; 45dgr, 45° direction; 135dr, 135° direction.

**Table 2:** Univariate analysis of texture parameters. Values are expressed as mean ± standard deviation.

Feature	Hemorrhagic Transformation	No Hemorrhagic Transformation	*p*-Value	Raw *p*-Value	FDR-Adjusted *p*-Value
135dr_RLNonUni	1210.85 ± 622.95	2867.40 ± 768.49	<0.001	5.31 × 10^−7^	8.85 × 10^−7^
45dgr_RLNonUni	1207.10 ± 629.40	2859.52 ± 766.79	<0.001	5.31 × 10^−7^	8.85 × 10^−7^
Vertl_GLevNonU	114.06 ± 54.01	283.58 ± 93.64	<0.001	1.45 × 10^−7^	3.63 × 10^−7^
Horzl_RLNonUni	1081.95 ± 579.52	2605.35 ± 736.35	<0.001	6.68 × 10^−7^	9.54 × 10^−7^
Horzl_GLevNonU	116.87 ± 55.20	291.16 ± 100.40	<0.001	1.29 × 10^−7^	3.63 × 10^−7^
45dgr_GLevNonU	122.44 ± 57.53	304.04 ± 105.79	<0.001	1.14 × 10^−7^	3.63 × 10^−7^
135dr_GLevNonU	122.89 ± 57.75	303.72 ± 105.16	<0.001	1.01 × 10^−7^	3.63 × 10^−7^
Vertl_RLNonUni	1038.01 ± 567.19	2458.63 ± 703.23	<0.001	9.39 × 10^−7^	1.17 × 10^−6^
S(4, 0)Correlat	0.087 ± 0.121	0.006 ± 0.081	0.026	0.0261	0.0290
S(3, 0)Correlat	0.106 ± 0.141	0.014 ± 0.080	0.041	0.0406	0.0406

**Table 3:** Receiver operating characteristic analysis of texture parameters, including area under the curve values with 95% confidence intervals.

Feature	AUC	Cut-Off	Sensitivity (%)	Specificity (%)
135dr_RLNonUni	0.95 (0.89–1.00)	≤1776.00	86.7	94.1
45dgr_RLNonUni	0.95 (0.89–1.00)	≤1462.45	80.0	100.0
Vertl_GLevNonU	0.97 (0.92–1.00)	≤180.76	90.0	100.0
Horzl_RLNonUni	0.94 (0.88–1.00)	≤1735.76	90.0	88.2
Horzl_GLevNonU	0.97 (0.92–1.00)	≤191.56	90.0	100.0
45dgr_GLevNonU	0.97 (0.93–1.00)	≤203.16	90.0	100.0
135dr_GLevNonU	0.97 (0.92–1.00)	≤207.37	93.3	100.0
Vertl_RLNonUni	0.94 (0.87–1.00)	≤1132.57	76.7	100.0
S(4, 0)Correlat	0.30 (0.15–0.45)	≤−0.11	6.7	94.1
S(3, 0)Correlat	0.32 (0.16–0.47)	≤−0.10	6.7	94.1

## Data Availability

The data presented in this study are available on request from the corresponding author due to privacy reasons.

## Abbreviations

The following abbreviations are used in this manuscript:

135dr	135° Direction
45dgr	45° Direction
AIS	Acute Ischemic Stroke
ASPECTS	Alberta Stroke Program Early CT Score
AUC	Area Under the Curve
CI	Confidence Interval
CT	Computed Tomography
ECASS	European Cooperative Acute Stroke Study
F	Fisher Coefficient
GLevNonU	Gray-Level Non-Uniformity
HAT	Hemorrhage After Thrombolysis
HI	Hemorrhagic Infarction
Horzl	Horizontal Direction
HT	Hemorrhagic Transformation
NCCT	Non-Contrast Computed Tomography
PH	Parenchymal Hematoma
RLNonUni	Run-Length Non-Uniformity
ROC	Receiver Operating Characteristic
ROI	Region of Interest
S(3, 0)Correlat	Gray-Level Co-occurrence Matrix Correlation (offset 3, 0)
S(4, 0)Correlat	Gray-Level Co-occurrence Matrix Correlation (offset 4, 0)
SEDAN	Blood Sugar, Early Infarct Signs, Hyperdense Artery Sign, Age, and National Institutes of Health Stroke Scale
Vertl	Vertical Direction
tPA	Tissue Plasminogen Activator
